# Altered Expression of Transfer-RNA-Derived Small RNAs in Human With Rheumatic Heart Disease

**DOI:** 10.3389/fcvm.2021.716716

**Published:** 2021-12-01

**Authors:** Zhao-yu Yang, Peng-fei Li, Zhi-qing Li, Tao Tang, Wei Liu, Yang Wang

**Affiliations:** ^1^Department of Integrated Traditional Chinese and Western Medicine, Institute of Integrative Medicine, Xiangya Hospital, Central South University, Changsha, China; ^2^National Clinical Research Center for Geriatric Disorders, Xiangya Hospital, Central South University, Changsha, China; ^3^Department of Respiratory and Critical Care Medicine, The First Affiliated Hospital of Zhengzhou University, Zhengzhou, China; ^4^Hunan University of Chinese Medicine, Changsha, China; ^5^Department of Cardiovascular Surgery, Xiangya Hospital, Central South University, Changsha, China

**Keywords:** rheumatic heart disease, atrial fibrillation, transfer RNA derived small RNAs, transcriptomics, biomarker

## Abstract

Rheumatic heart disease (RHD) remains a severe public health problem in developing countries. Atrial fibrillation (AF) is a medical complication of RHD. Although the understanding of disease pathogenesis has advanced in recent years, the key questions need to be addressed. Transfer RNA–derived small RNAs (tsRNAs) are a novel type of short non-coding RNAs with potential regulatory functions in various physiological and pathological processes. The present study used tsRNAs sequencing to investigate the relationship between RHD and atrial fibrillation (AF). Three paired cardiac papillary muscles were taken from six rheumatic RHD patients with AF (3 cases) or without AF (3 cases) from January 2016 to January 2017 in Xiangya Hospital, Central South University. A total of 219 precisely matched tsRNAs were identified, and 77 tsRNAs (fold change > 2.0 and *P* < 0.05) were differently changed. Three tsRNAs (AS-tDR-001269, AS-tDR-001363, AS-tDR-006049) were randomly selected and confirmed by qRT-PCR. The results of qRT-PCR were consistent with tsRNAs sequencing, suggesting the tsRNAs sequencing was reliable. Subsequently, we predicted the target mRNAs of the three tsRNAs. Moreover, we verified the functions of tsRNAs targeting mRNAs *in vitro*. Finally, bioinformatics analysis indicated that the target genes were abundant in regulation of transcription, DNA binding, intracellular. Most of the genes were predicted to interplay with cytokine-cytokine receptor by KEGG analysis. Our findings uncover the pathological process of AF in RHD through tsRNAs sequencing. This research provides a new perspective for future research on elucidating the mechanism of AF in RHD and offers potential new candidates for the treatment and diagnosis.

## Introduction

Rheumatic heart disease (RHD) is a chronic autoimmune valvulitis, resulting from an autoimmune response to a group A streptococcal infection (1, 2). RHD remains a neglected disease and is a major cause of morbidity and mortality in many developing countries (2, 3). It is currently estimated that 40.5 million individuals around the world live with RHD (4). There were 306,000 deaths due to RHD during 2019 worldwide (4). Atrial fibrillation (AF) is a medical complication of RHD. AF occurs in approximately one-fifth of patients with RHD (5, 6). However, the pathogenesis of AF in RHD and the underlying signaling pathways are still poorly understood.

In recent years, a growing number of evidence indicated that the small non-coding RNAs play important roles in the pathophysiological mechanism of AF (7, 8). With the advent of multiple high-throughput sequencing technologies, numerous novel classes of small RNA have emerged. New classes of “non-micro-short” RNAs named transfer-RNA-derived small RNAs (tsRNAs, <50 nucleotides) attracted our attention (9–17). tsRNAs are the second abundant class of small non-coding RNAs (18) and can regulate biological processes, such as proliferation, apoptosis, and epigenetic inheritance (19, 20). tsRNAs are abundant small ncRNAs that account for 4–10% of all cellular RNA (21). Generally, tsRNAs are divided into two main types, tRNA-derived fragment (tRF)s and tRNA-derived stress-induced RNA (tiRNA)s, based on their length and cleavage sites. tsRNAs regulate gene expression by directly inhibiting protein synthesis (22) or acting as the guide RNA in a miRNA-fashion (14). Multiple innovative investigations have divulged that dysregulated tsRNAs are closely related to human diseases, such as neurological disorders (23), metabolic disorders (24), and cancer (25). Emerging evidence has proved that tsRNAs are detected in the heart. The cardiac pathophysiological conditions could be induced to tsRNAs biogenesis (26). A new study revealed that tsRNAs were potential therapeutic targets to cure myocardial ischemic injury (27). However, the expression of tsRNAs of RHD with AF is never discussed. Therefore, we intended to unveil the potential pathological mechanism of AF in RHD via tsRNAs. In addition, tsRNAs will provide novel approaches in grasping new therapeutic targets and understanding the underlying mechanisms of AF in RHD.

In the present study, we discovered the distinct difference in the expression of tsRNAs between RHD with AF and RHD without AF. Bioinformatics analysis identified potential targets genes and evaluated the putative biological functions. Our findings elucidate the molecular mechanism underlying RHD with AF and advance the knowledge of AF, which is of great clinical significance.

## Materials and Methods

### Patients Population

From January 2016 to January 2017, patients with RHD undergoing mitral valve replacement surgery (MVR) were enrolled from Xiangya Hospital, Central South University, Changsha, China. Written informed consents were acquired from all patients, and the study was passed by the ethics committee of the hospital and following the relevant guidelines and regulations. The number of Ethical Review is 201512546. Cardiac papillary muscles were obtained from patients who exhibited clinical characteristics of RHD with AF (*n* = 3) and without AF (*n* = 3). AF group: patients had permanent AF (documented arrhythmia more than 6 months) with mitral valve stenosis. The exclusion criteria contained a history of using anti-arrhythmic medications in the past 6 months, myocardial infarction, ischemic cardiomyopathy, heart failure, other types of arrhythmias, chronic hepatic or renal failure, and diabetes. The cardiac papillary muscles were immediately frozen in liquid nitrogen after surgical excision and stored at −80°C before sequencing.

### RNA Extraction

The total RNA was isolated from cardiac papillary muscles. Briefly, the tissues were homogenized with an electric homogenizer after adding TRIzol (Invitrogen life technologies). Then, Chloroform was added and centrifuged at 12,000 × g for 15 min to dissolve the RNA in the aqueous phase. Adding isopropanol to make RNA precipitated, and the resultant RNA pellet was then washed with 75% ethanol and dissolved in RNase-free water. Using NanoDrop ND-1000 identified the quality and concentration of RNA. The total optical densities at a 260/280 nm absorbance ratio of all total RNA samples ranged from 1.8 to 2.0. All RNA solutions were then stored at −80°C.

### tsRNAs Sequencing

The total RNA of the tissues for sequencing was pretreated to remove some RNA modifications. The following experiments were carried out to remove some RNA modifications that may disturb small RNA-sequencing library construction (28, 29): 3′-aminoacyl (charged) deacylation to 3′-OH (hydroxyl group) for 3′ adaptor ligation, 3′-cP (2′, 3′-cyclic phosphate) removal to 3′-OH for 3′ adaptor ligation, 5′-OH phosphorylation to 5′-P for 5′-adaptor ligation, and N1-methyladenosine and N3-methylcytidine demethylation for efficient reverse transcription. All methods were conducted based on the rtStar tRF&tiRNA Pretreatment Kit (Arraystar, USA) protocols. The Shanghai BioChip Company constructed the small RNA library and carried out the Solexa high-throughput sequencing following their standard protocols. Briefly, the total RNA of each sample was sequentially ligated to 3′ and 5′ small RNA adapters. cDNA was then synthesized and amplified on Illumina's proprietary RT primers and amplification primers. Subsequently, ~135–160 bp PCR amplified fragments were extracted and purified from the PAGE gel. The purified libraries were qualified with the NanoDrop™ ND-1000 Fluorometer (Thermofisher, ND-1000, German) and validated using the Agilent 2100 bioanalyzer (Agilent, G2938C, USA) to verify the insert size and figure out the molar concentration. Only the library that passed quality control was sequenced on an Illumina NextSeq 500/550 V2 kit (#FC-404–2005, Illumina, San Diego, CA, USA). Sequencing was carried out by running fifty cyclings.

### Data Processing and Analysis

Sequencing quality was examined by FastQC software, and trimmed reads (pass Illumina quality filter, trimmed 3′-adaptor bases by cut adapt) were aligned to mature-tRNA and pre-tRNA sequences from GtRNAdb (http://gtrnadb.ucsc.edu/) using NovoAlign software (v2.07.11). Only exactly matched reads were selected as tsRNAs. Moreover, tsRNAs expression levels were calculated and normalized as tag counts per million of total aligned tRNA reads (TPM). The expression profiling and differential expression analysis of tsRNAs were measured by the average TPM. The expression profiling and differential expression of tRNAs were calculated based on fold-change > 2.0 and *P* < 0.05 normalized TPM (30). Hierarchical clustering and volcano plots were conducted in the differentially expressed tsRNAs in the R environment for statistical computing and graphics.

### Small RNA Real-Time Quantitative PCR

tsRNAs were reverse transcription with specific primers (Table 2) using rtStar™ First-Strand cDNA Synthesis Kit (3′ and 5′ adaptor; Arraystar) following the manufacturer's instructions. Realtime-qPCR (qRT-PCR) amplification was performed using the ViiA 7 Real-time PCR System (Applied Biosystems) and 2 × PCR master mix (Arraystar). The cycling conditions (95°C, incubation, 10 min; 95°C, 40 cycles,10 s; 60°C, 60 s; and 95°C, 15 s). U6 was used for normalization. Using the 2^−ΔΔCt^ method to calculate the relative tsRNA expression levels. qRT-PCR reactions for all samples were performed in triplicate.

**Table 1:** The details of 14 variant tsRNAs in the RHD without AF and RHD with AF (fold change > 2 and *P* < 0.05).

**tDRs_ID**	**Type**	**Fold change (B/A)**	***P*** **_value**
AS-tDR-000123	tRF-1	−4.78	0.020
AS-tDR-007326	tRF-1	−4.46	0.030
AS-tDR-000102	tRF-3	−4.41	0.001
AS-tDR-007245	tRF-1	−4.31	0.018
AS-tDR-007294	tRF-1	−4.27	0.029
AS-tDR-000886	tiRNA-5	−4.11	0.041
AS-tDR-000894	tiRNA-5	−3.80	0.025
AS-tDR-000205	tiRNA-3	−3.72	0.005
AS-tDR-006049	tRF-3	−3.40	0.003
AS-tDR-001363	tiRNA-5	−3.30	0.006
AS-tDR-001297	tiRNA-5	2.10	0.004
AS-tDR-001269	tiRNA-5	2.55	0.048
AS-tDR-001270	tiRNA-5	2.23	0.023
AS-tDR-001289	tiRNA-5	2.28	0.037

*tDR, tRNA-derived small RNA; tRF, transfer RNA-derived fragment; tiRNA, tRNA halves; A, RHD without AF; B, RHD with AF*.

**Table 2:** Sequences of primers for qPCR validation.

**Name**	**Sequence**	**Product length (bp)**
U6	F:5′ GCTTCGGCAGCACATATACTAAAAT 3′ R:5′ CGCTTCACGAATTTGCGTGTCAT 3′	89
AS-tDR-001363	F:5′ ATCGCCCGGCTAGCTCAGT 3′ R:5′ TTCCGATCTAGAGTCCCATGCTC 3′	47
AS-tDR-006049	F:5′ TTCTACAGTCCGACGATCATCT 3′ R:5′ TCTTCCGATCTTGGAGGTTC 3′	47
AS-tDR-001269	F:5′ACAGTCCGACGATCTCCCATA 3′ R:5′ TCTAAAACCAGGAATCCTAACCG3′	52
GAPDH	F: 5′ ACAGCCTCAAGATCATCAGC 3′ R: 5′ GGTCATGAGTCCTTCCACGAT 3′	89
TNFRSF1B	F: 5′CGGCTCAGAGAATACTATGACC 3′ R: 5′ACAGAAGACTTTTGCATGTTGG 3′	81
CCL5	F: 5′AGAGCTGCGTTGCACTTGTT 3′ R: 5′GCAGTTTACCAATCGTTTTGGGG 3′	84

### Cell Culture and Transfection

AC16 cells (Zhongqiaoxinzhou Biotech, shanghai, Chian) were cultured in Dulbecco's modified Eagle's medium (DMEM; Gibco, USA) containing 10% of fetal bovine serum (Gibco, USA) and incubators at 37°C. Then AC16 cells were cultured into 12-well plates for transfection. The AS-tDR-001363 mimic (sense: 5′ GCCCGGCUAGCUCAGUCGGUAGAGCAUGGGACUCU 3′, antisense: 5′ CGGGCCGAUCGAGUCAGCCAUCUCGUACCCUGAGA 3′) was obtained from RiboBio (Guangzhou, China). The final concentration of transfection of mimics and NC was 100 nM. Using Lipofectamine 3000 (Invitrogen, USA) to help transfect mimics and NC based on the manufacturer's instructions. All experiments were performed in triplicate. The total RNA was isolated from the transfected cells. The tsRNA-targeted genes were then measured by qRT-PCR. The specific primers were listed in Table 2, and the protocols were described as above.

### Target Prediction and Bioinformatics

tsRNAs could target mRNA leading to mRNA degradation in a microRNA (miRNA) manner. Here we used two common algorithms to predict tsRNA targets, namely, TargetScan v6.0 (http://www.targetscan.org) and miRanda (http://www.microrna.org) (31, 32). The overlapping target genes were applied to further bioinformatics. The biological process of the target genes was conducted by Gene Ontology (GO) annotations and Kyoto Encyclopedia of Genes and Genomes (KEGG) pathway analysis, through DAVID Bioinformatics Resources 6.8 (https://david.ncifcrf.gov) (33). Cytoscape software (version 3.7.2, the Cytoscape Consortium, San Diego, CA, USA) was used to construct the network.

### Statistical Analysis

Data are presented as mean ± standard error. Two-group difference analysis was used Student's *t*-test. The limma package in R software (version 4.0.4) was applied to determine the differential expression of tsRNAs and the pheatmap package in R was used to construct heat map. The ggplot2 built the figure of heat map. Fold change > 2.0 and *P* < 0.05 were considered to indicate a statistically significant difference in sequence analysis.

## Results

### Expression Profiles of tsRNAs

**Figure 1:**
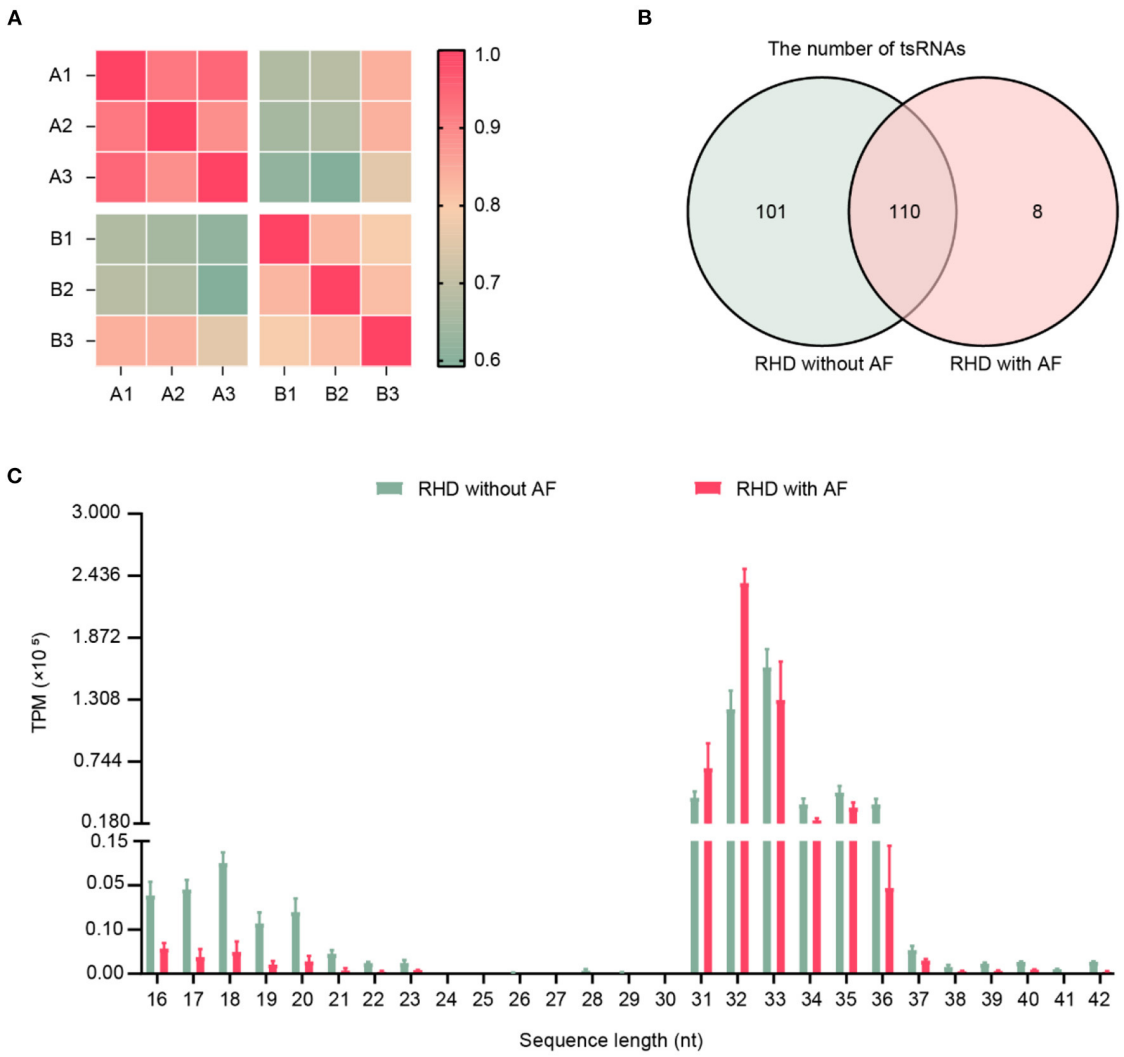
Expression profiles of tRFs/tiRNAs sequencing data in RHD with AF and RHD without AF. **(A)** The correlation coefficient was applied to evaluate the criterion of reliability, and it is reasonable for the sample selection (RHD without AF: A1, A2, A3; RHD with AF: B1, B2, B3). **(B)** Venn plot displayed the total number of identified tsRNAs in RHD with the AF group and RHD without the AF group. **(C)** Length distributions of tsRNA in the RHD without AF and RHD with AF (TPM: tsRNA expression levels normalized as tag counts per million of total aligned tRNA reads).

The clinical characteristics of patients were summarized in Table 3, including age, gender, and color doppler echocardiography. To explore the involvement of small RNAs in RHD patients, the cardiac papillary muscles of the patients were processed for tsRNAs sequencing (tsRNA-seq). The RNA-seq data have been deposited into GEO (GSE185581). The correlation analysis was based on the TPM counts of each sample. The correlation coefficient is a vital evaluation criterion of the reliability and reasonability of the sample selection (34, 35). As shown in Figure 1A, the correlation coefficient of the compared samples in the same group was more than 0.9 and in the different groups was <0.75. That is to say, a distinguishable tsRNAs expression profiling was found among the two groups. A total of 219 tsRNAs were identified (211 in RHD without AF, 118 in RHD with AF) (Figure 1B). The tsRNAs of the tsRNAs' distributed at the length of 16–21 and 31–37 nt. RHD with AF group compared to RHD without AF group, the content of tsRNAs with different lengths was changed (Figure 1C).

**Table 3:** The general condition of patients between the two groups.

	**RHD without AF**	**RHD with AF**
	**(*n* = 3)**	**(*n* = 3)**
Age (year)	48.67 ± 0.58	54.68 ± 15.04
Sex (M/F)	2/1	2/1
LA (mm)	37.33 ± 7.51	45 ± 8.19
RA (mm)	43.33 ± 4.04	48.33 ± 9.50
LV (mm)	42.33 ± 1.53	49.00 ± 3.00
EF (%)	59.67 ± 1.53	55.67 ± 1.34

*M, male; F, female; LA, left atrium; RA, Right atrium; LV, left ventricular; EF, ejection fraction*.

### Changes of tsRNAs Expression

**Figure 2:**
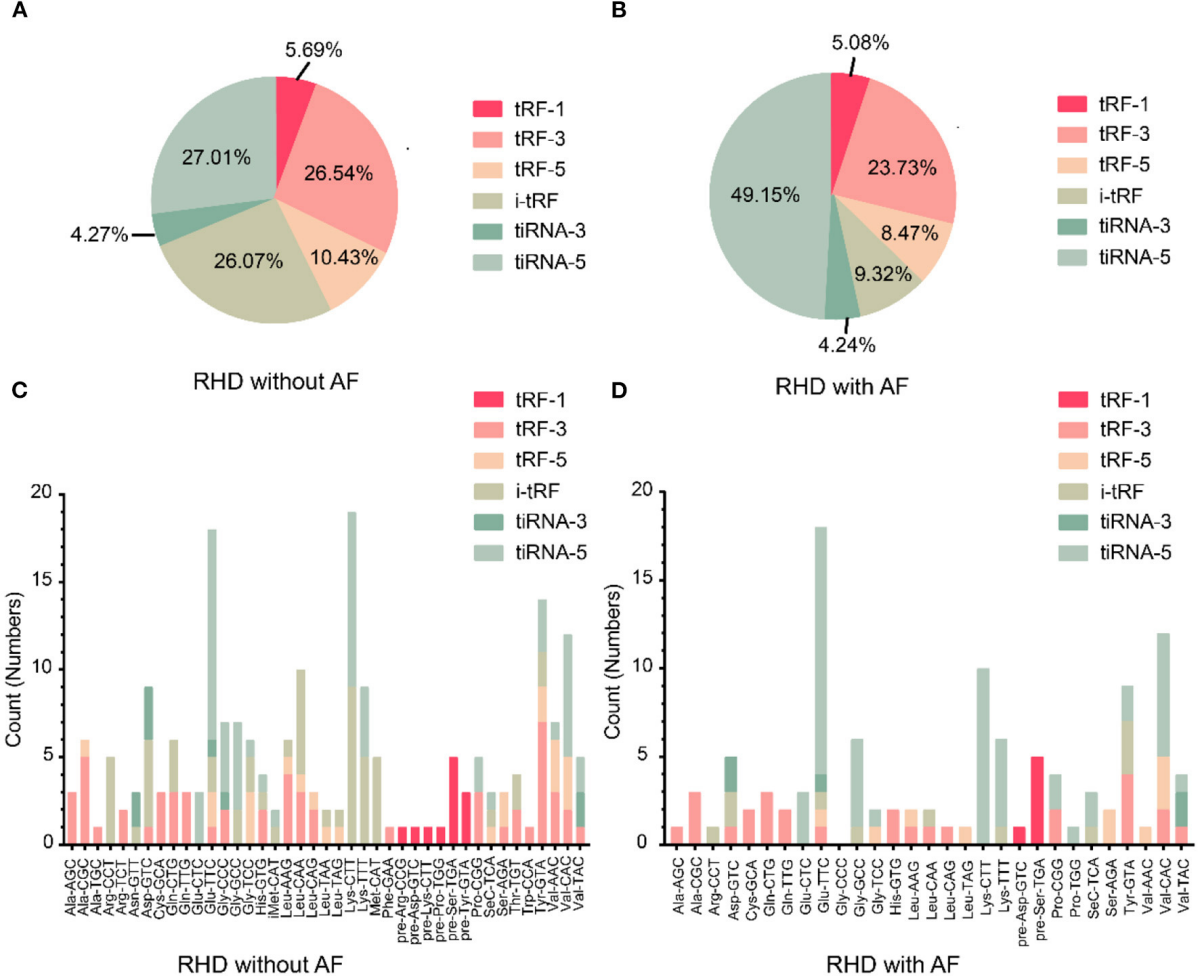
Differences and characterizations of tsRNAs expression profiles between two groups. **(A,B)** Pie chart of the distribution of subtypes of tsRNAs numbers in RHD without AF **(A)** and RHD with AF **(B)**. **(C,D)** Stacked plot for all subtypes of tsRNAs of each group clustering by the same anticodon of the tRNAs in RHD without AF **(C)** and RHD with AF **(D)**.

To investigate whether tsRNAs types were altered, we estimated the subtype numbers of tsRNA transcripts in both RHD without AF and RHD with AF groups. Over 99% of tsRNAs were originated from mature tsRNAs (tRF-1, tRF-3, tRF-5, i-tRF, tiRNA-3, tiRNA-5). Further analysis showed that most tsRNAs in both groups were tiRNA-5, and the proportion of RHD without AF and RHD with AF was 27.01 and 49.15%, respectively (Figures 2A,B). The pie chart demonstrated that the RHD with AF group mainly increased the expression of tiRNA-5 and decreased the expression of other tsRNAs (Figures 2A,B). In addition, the numbers of tsRNAs derived from the variable anticodon tRNAs are demonstrated in the stacked plots (Figures 2C,D). All results suggested that the types of tsRNAs were different in RHD without AF and RHD with AF groups.

### Identification of Related tsRNAs and qRT-PCR Confirmation

**Figure 3:**
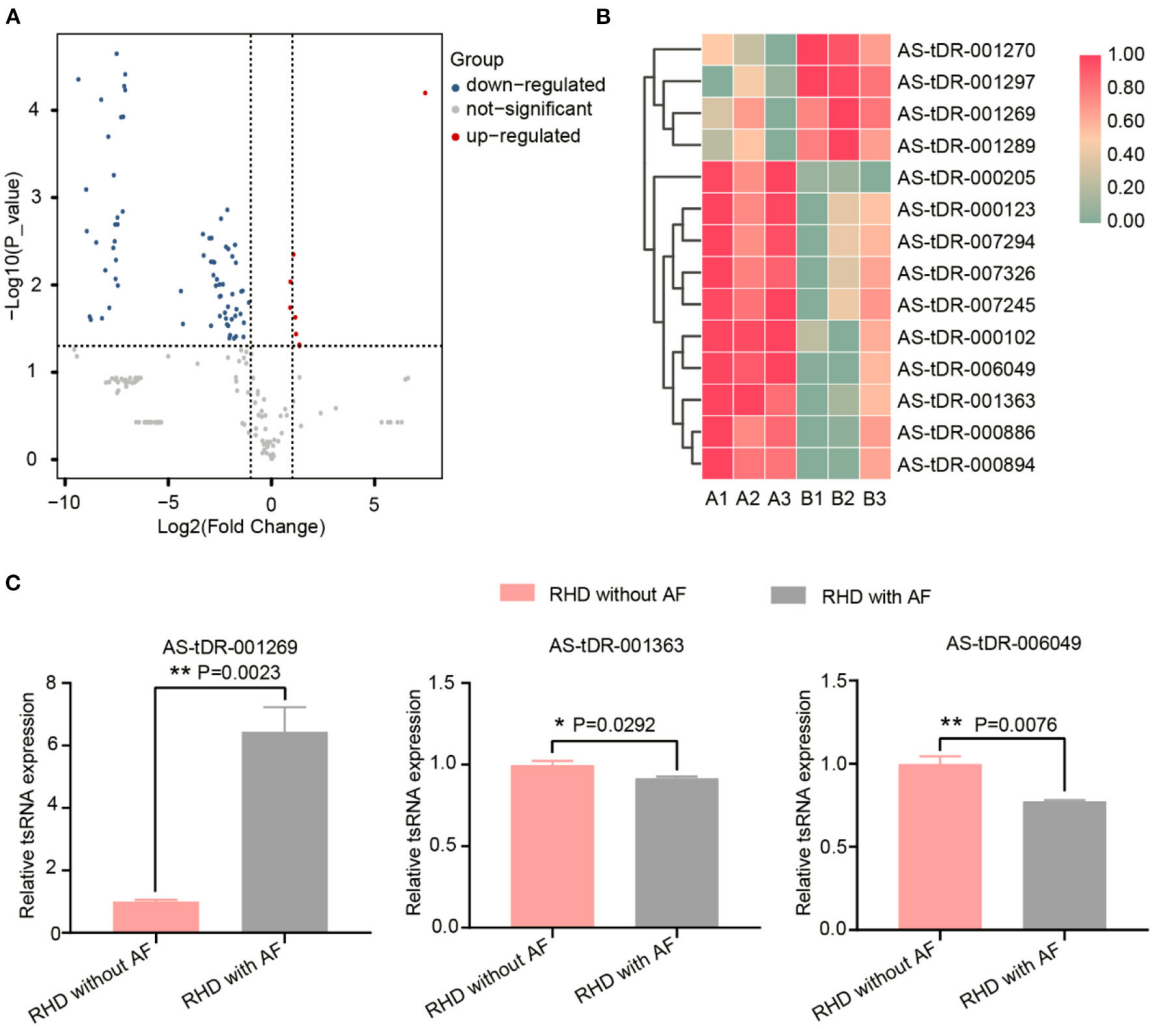
The dysregulated tsRNAs expression profiles and the relative expression of selected tsRNAs were confirmed by qRT-RCR. **(A)** Volcano maps of differentially expressed tsRNAs. The volcano plot's X and Y axes have values of log_2_ (Fold Change) and –log_10_ (*P*_value). With a fold change > 2 and a *P* < 0.05, red/blue dots indicate statistically considerable differentially expressed tsRNAs (red depicts elevated expression while blue indicates decreased expression). No differentially expressed tsRNAs are indicated by gray dots. **(B)** The hierarchical clustering heat-map for the 14 aberrantly expressed tsRNAs (RHD without AF: A1, A2, A3; RHD with AF: B1, B2, B3). **(C)** The qRT-PCR results were consistent with the RNA-Seq data. AS-tDR-001269 (*P* = 0.0023), AS-tDR-001363 (*P* = 0.0292), AS-tDR-006049 (*P* = 0.0076) were statistically different between RHD with the AF group and RHD without the AF group. Data were present as mean ± SEM (*n* = 3 for each group). *P < 0.05 represent RHD with AF compared to RHD without AF; **P < 0.01 indicated RHD with AF compared to RHD without AF. qRT-PCR, quantitative real-time PCR; RNA-Seq, RNA sequencing.

We looked at changes in expression for the individual tsRNAs using a standard of fold change > 2.0 and P < 0.05 for significant changes in expression. A total of 77 tsRNAs were differentially expressed in the RHD with AF group compared to RHD without AF group. The volcano plot showed six tsRNAs up-regulated and 71 tsRNAs down-regulated (Figure 3A). Fourteen tsRNAs (4 up-regulated: AS-tDR-001270, AS-tDR-001297, AS-tDR-001269, and AS-tDR-001289; 10 down-regulated: AS-tDR-000205, AS-tDR-000123, AS-tDR-007294, AS-tDR-007326, AS-tDR-007245, AS-tDR-000102, AS-tDR-006049, AS-tDR-001363, AS-tDR-000886, AS-tDR-000894) were significantly altered in RHD with the AF group compared with RHD without AF group (Figure 3B, Table 1). We constructed a hierarchical clustering map to examine these differentially expressed tsRNAs. The RHD with the AF group clustered together in one group were primarily distinct from the RHD without the AF group (Figure 3B). We used qRT-PCR to confirm the expression changes for the three tsRNAs (AS-tDR-001269, AS-tDR-001363, and AS-tDR-006049). The expression level of AS-tDR-001269 (*P* = 0.0023) was up-regulated with the statistical difference between RHD with the AF group and RHD without the AF group. The expression levels of AS-tDR-001363 (*P* = 0.0292) and AS-tDR-006049 (*P* = 0.0076) in RHD with the AF group were down-regulated (*P* < 0.05), compared to RHD without AF group (Figure 3C). The qRT-PCR results of AS-tDR-001269, AS-tDR-001363, and AS-tDR-006049 were consistent with the RNA-seq, indicating that the results had higher reliability.

### Prediction of Target Genes of tsRNAs and Validation of Target Genes

**Figure 4:**
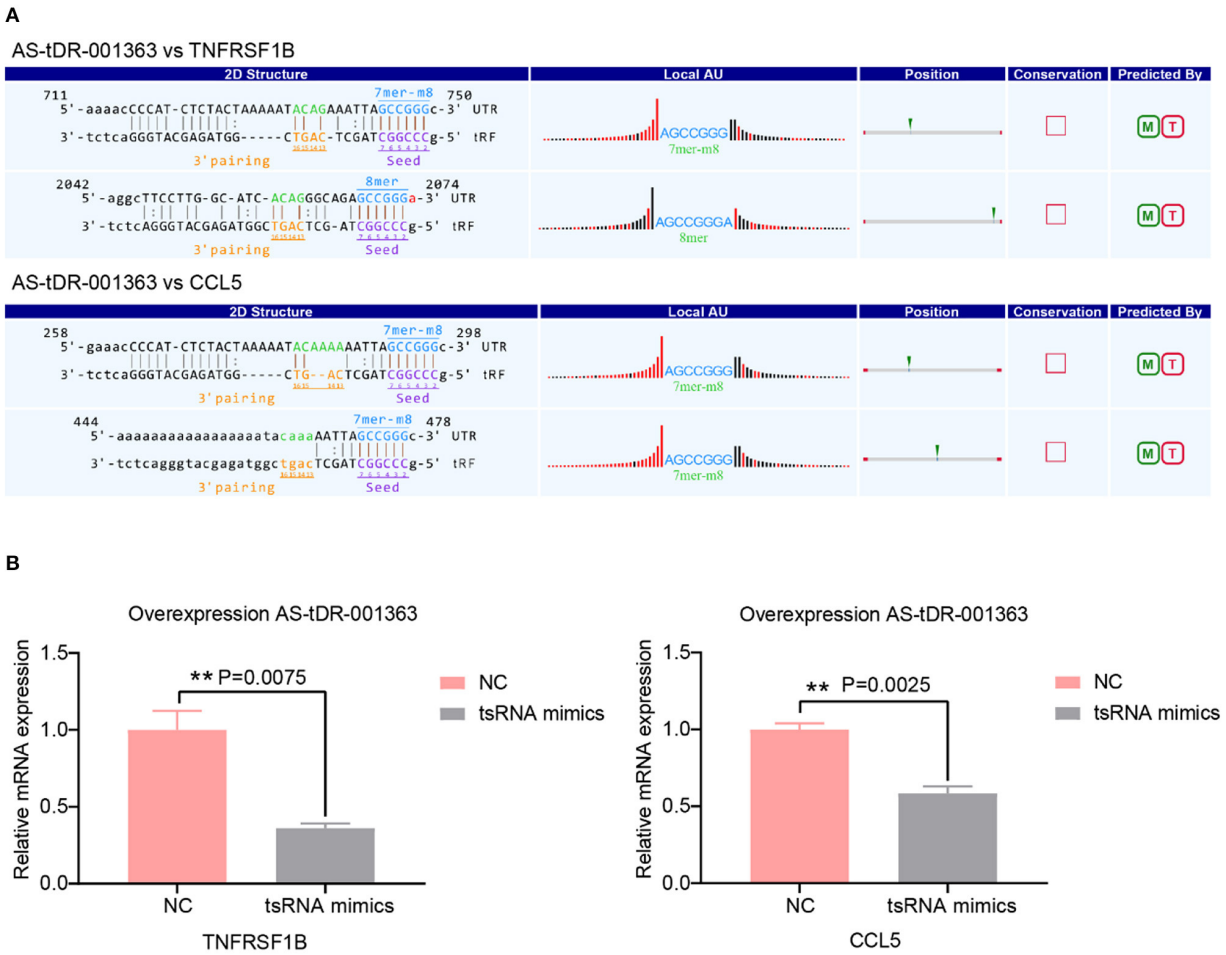
Target genes validation. **(A)** The binding region and seed sequence of AS-tDR-001363 randomly selected mRNA transcripts (TNFRSF1B and CCL5). **(B)** The relative mRNAs levels detected by qRT-PCR in AC16 cells transfected with tsRNAs mimics. The qRT-PCR results of TNFRSF1B and CCL5 level in AC16 cells transfected with AS-tDR-001363. The data are exhibited as the mean ± SEM (*n* = 3). **P < 0.01 presented tsRNAs mimics compared to the NC group.

A growing number of evidence has revealed that tsRNAs contain some seed sequences that might match the seed regions of mRNA by antisense pairing, regulating the expression level of the target mRNA (16, 23, 36). Although different algorithms can get possible seed sequences and targets for tsRNAs, each methodology for tsRNA target prediction is referenced to miRNA target predictors (23). Therefore, the sequences of altered three tsRNAs were loaded to TargetScan and miRanda to acquire the targets genes. According to the above theory, miRanda and Targetscan, two algorithms were used for predicting the target genes. In total, 3,123 mRNA targets were predicted simultaneously for the three validated tsRNAs (Supplementary Figure 1). The target genes of AS-tDR-001269, AS-tDR-001363, and AS-tDR-006049 were 1,861, 1,179, and 336, respectively. In the current study, AS-tDR-001363 was reduced in the RHD with the AF group. The qRT-PCR was used to verify the relationship between tsRNAs and their relative mRNAs. The two mRNA genes (TNFRSF1B and CCL5)-the target genes of AS-tDR-001363-were selected. The binding site and seed sequence of these tsRNAs and their target mRNAs are displayed in Figure 4A. To confirm the relationship between the target genes and tsRNAs, we overexpressed AS-tDR-001363 in AC16 cells to identify the corresponding alterations in tsRNA target genes. After transfection with AS-tDR-001363 mimics, the expression of TNFRSF1B and CCL5 were predominantly downregulated (Figure 4B). The qRT-PCR results of *in vitro* experiments explain the relationship between tsRNAs and target mRNAs. Therefore, the forecasted targets could be applied to further analysis.

### Biological Function Analysis

**Figure 5:**
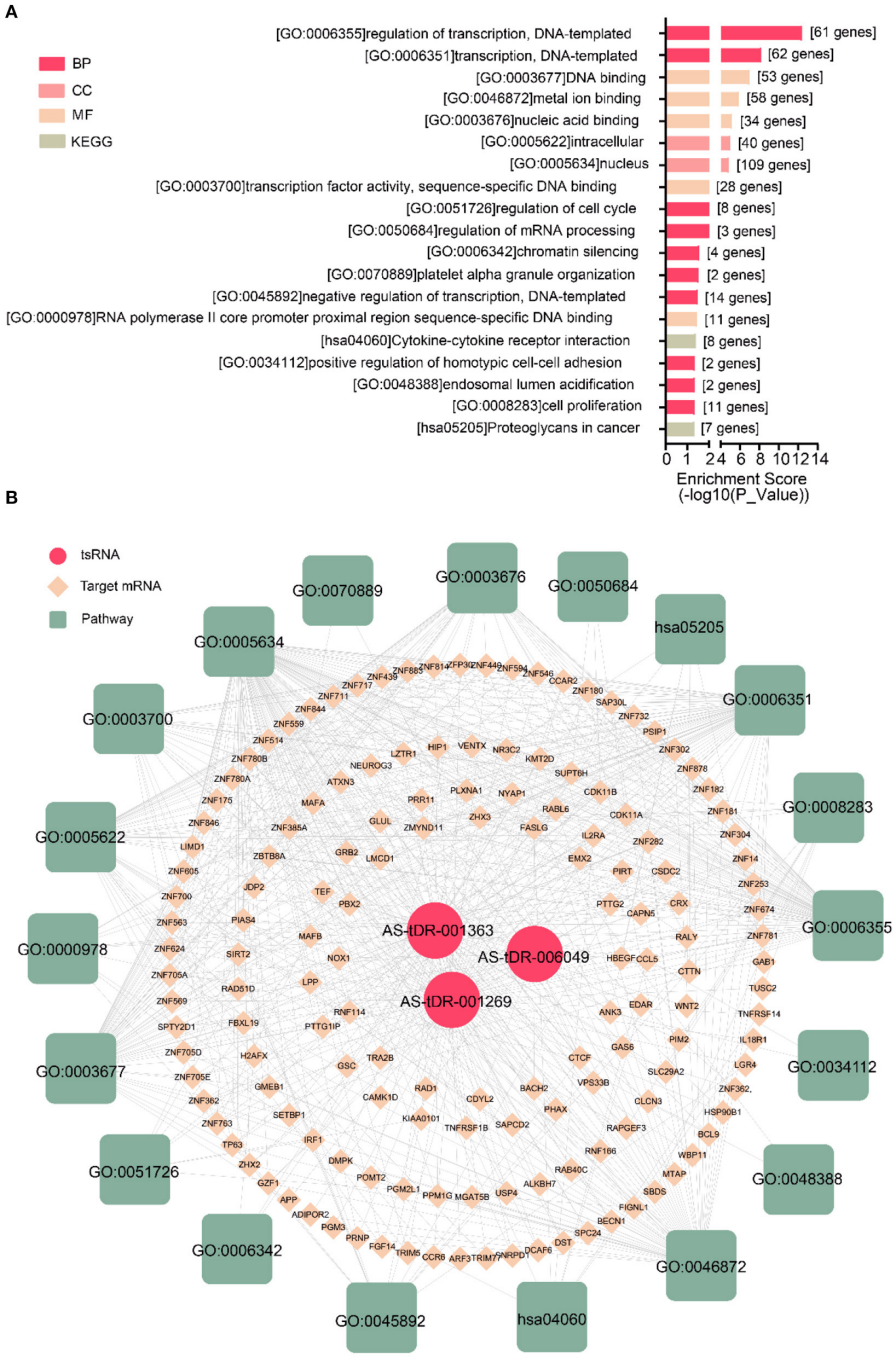
Biological annotation of targets to reveal the function of altered tsRNAs. **(A)** The top 19 enriched terms were shown ranked by *P*_value. **(B)** The interaction networks of tsRNA-mRNA-pathway.

We performed a bioinformatics analysis of the target genes with a context <−0.4. GO biological processes and KEGG pathway enrichment analysis was executed to explore the functions of 278 target genes by using the DAVID online analysis tool (Figure 5B). GO analysis included molecular function (MF), biological processes (BP), and cell composition (CC). The primary biological processes observed by GO were regulation of transcription (BP; GO: 00060355; 61 genes), DNA binding (MF; GO: 0003677, 53 genes), intracellular (CC; GO: 0005622, 40 genes), etc (Figure 5A, Table 4). According to KEGG enrichment analysis, cytokine-cytokine receptor interaction (hsa04060; 8 genes) and proteoglycans in cancer (has05205; 7 genes) were significantly detected (Figure 5A, Table 4).

**Table 4:** The significant enriched GO and KEGG pathways of target genes.

**ID**	**Term**	**Count**	**Genes**	***P*** **_value**
GO:0006355	Regulation of transcription, DNA-templated	61	RALY, ZNF781, ZNF674, PTTG2, ZNF253, ZNF14, CRX, ZNF304, ZNF181, ZNF182, ZNF878, ZNF302, PSIP1, ZNF732, SAP30L, ZNF180, CCAR2, CSDC2, ZNF546, ZNF594, ZNF282, ZNF440, ZFP30, EMX2, ZNF814, ZNF883, ZNF439, CDK11A, ZNF717, ZNF711, CDK11B, SUPT6H, ZNF844, KMT2D, ZNF559, NR3C2, VENTX, ZNF514, ZNF780B, ZNF780A, ZNF175, ZNF846, LIMD1, ZNF605, ZNF700, HIP1, ZNF563, ZNF624, LZTR1, ZNF705A, ZNF569, SPTY2D1, ZNF705D, ZNF705E, NEUROG3, ATXN3, ZNF362, ZNF763, MAFA, ZNF385A, ZBTB8A	3.93481E-13
GO:0006351	Transcription, DNA-templated	62	RALY, JDP2, ZNF781, FASLG, TP63, ZNF674, ZNF253, ZNF14, ZNF304, ZNF181, ZNF182, ZNF878, ZNF302, PSIP1, ZNF732, SAP30L, ZNF180, CCAR2, ZNF546, ZNF594, ZNF282, ZNF440, ZFP30, ZHX2, ZHX3, GZF1, ZNF883, ZNF439, PIAS4, ZNF717, ZNF711, SUPT6H, ZNF844, KMT2D, ZMYND11, ZNF559, NR3C2, ZNF514, ZNF780B, ZNF780A, ZNF175, ZNF846, LIMD1, ZNF700, ZNF605, HIP1, ZNF563, ZNF624, ZNF705A, ZNF569, SPTY2D1, ZNF705D, LMCD1, ZNF705E, SIRT2, ATXN3, ZNF362, ZNF763, MAFA, PBX2, ZNF385A, ZBTB8A	5.91126E-09
GO:0003677	DNA binding	53	RAD51D, ZNF781, TP63, ZNF674, CRX, ZNF14, FBXL19, ZNF181, APP, ZNF182, ZNF302, H2AFX, SAP30L, ZNF180, CSDC2, ZNF546, ZNF594, ZNF282, ZNF440, ZFP30, GMEB1, ZHX2, ZHX3, ZNF883, ZNF439, PIAS4, ZNF717, ZNF711, SUPT6H, KMT2D, ZNF844, ZNF559, ZNF514, ZNF780B, ZNF780A, ZNF175, ZNF846, ZNF700, ZNF605, ZNF563, ZNF624, ZNF705A, SETBP1, ZNF569, SPTY2D1, ZNF705D, ZNF705E, ZNF362, IRF1, ZNF763, MAFA, ZNF385A, ZBTB8A	1.04414E-07
GO:0046872	Metal ion binding	58	ZNF781, TP63, ZNF674, ZNF253, ZNF14, DMPK, ZNF304, POMT2, ZNF181, ZNF182, ZNF878, ZNF302, ZNF732, SAP30L, ZNF180, ZNF546, ZNF594, ZNF282, ZNF440, ZFP30, GMEB1, ZNF814, ZHX2, ADIPOR2, ZHX3, PGM2L1, GZF1, ZNF883, PPM1G, PGM3, ZNF439, ZNF717, ZNF711, PRNP, ZNF844, MGAT5B, ZNF559, USP4, ZNF514, ZNF780B, ZNF780A, ZNF175, ZNF846, ZNF700, ZNF605, ALKBH7, ZNF563, ZNF624, ZNF705A, ZNF569, NOX1, ZNF705D, ZNF705E, RNF114, ZNF362, ZNF763, ZNF385A, ZBTB8A	1.22463E-06
GO:0003676	Nucleic acid binding	34	RALY, ZNF844, ZNF559, TRA2B, ZNF781, ZNF674, ZNF780B, ZNF253, ZNF514, ZNF780A, ZNF175, ZNF846, ZNF304, ZNF181, ZNF878, ZNF302, ZNF732, ZNF180, ZNF700, ZNF546, ZNF563, ZNF594, ZNF624, ZNF282, ZNF705A, ZNF440, ZFP30, ZNF569, ZNF814, ZNF439, ZNF717, ZNF763, ZNF385A, ZBTB8A	6.76917E-06
GO:0005622	Intracellular	40	ZNF844, ZNF559, FGF14, RAB40C, ZNF674, ZNF780B, ZNF514, ZNF780A, ZNF175, ZNF846, ZNF304, TRIM5, ZNF181, RNF166, ZNF302, ZNF732, RAPGEF3, ZNF180, ZNF700, ZNF546, ZNF563, ZNF624, CAPN5, PIRT, IL2RA, ZNF282, RABL6, ZNF705A, ZNF440, ZFP30, ZNF569, ZNF814, RNF114, ZNF439, CCR6, ARF3, ZNF717, ZNF763, NYAP1, TRIM77	9.71511E-06
GO:0005634	Nucleus	109	RAD51D, RALY, JDP2, PLXNA1, FGF14, PRR11, SNRPD1, ZNF781, PTTG2, ZNF253, ZNF304, ZNF181, ZNF182, ZNF302, PSIP1, H2AFX, SAP30L, ZNF180, CCAR2, ZNF594, ZNF440, EMX2, ZHX2, ZHX3, RAD1, DCAF6, ZNF439, GLUL, PIAS4, DST, SUPT6H, ZNF844, GRB2, VENTX, ZNF514, ZNF846, SPC24, CDYL2, TEF, HIP1, ZNF624, MAFB, LPP, BECN1, LMCD1, NEUROG3, PTTG1IP, MAFA, BACH2, FIGNL1, TP63, FASLG, CTCF, ZNF674, CRX, ZNF14, TRIM5, SBDS, ZNF878, ZNF732, CSDC2, ZNF546, GSC, ZNF282, RABL6, ZFP30, GMEB1, GZF1, ZNF883, PPM1G, CDK11A, ZNF717, ZNF711, MTAP, CDK11B, PRNP, CAMK1D, ZMYND11, KMT2D, ZNF559, TRA2B, USP4, NR3C2, KIAA0101, WBP11, ZNF780B, ZNF780A, TNFRSF1B, SAPCD2, LIMD1, ZNF700, ZNF605, BCL9, ZNF563, ZNF705A, SETBP1, ZNF569, ZNF705D, ZNF705E, SIRT2, PHAX, HSP90B1, RNF114, ATXN3, ZNF362, IRF1, ZNF763, PBX2, ZBTB8A	1.31622E-05
GO:0003700	Transcription factor activity, sequence-specific DNA binding	28	JDP2, BACH2, NR3C2, TP63, CTCF, ZNF780B, ZNF514, ZNF780A, ZNF175, CRX, ZNF304, ZNF182, ZNF302, ZNF605, ZNF546, ZNF624, LZTR1, ZFP30, ZHX2, ZNF814, ZHX3, ZNF883, ZNF717, IRF1, ZNF711, MAFA, PBX2, SUPT6H	8.31122E-04
GO:0051726	Regulation of cell cycle	8	RAD51D, FIGNL1, CDK11A, PRR11, KIAA0101, IRF1, CDK11B, SIRT2	2.34309E-03
GO:0050684	Regulation of mRNA processing	3	CDK11A, CDK11B, SUPT6H	7.19008E-03
GO:0006342	Chromatin silencing	4	KMT2D, H2AFX, SIRT2, SUPT6H	2.80589E-02
GO:0070889	Platelet alpha granule organization	2	VPS33B, ZNF385A	2.90859E-02
GO:0045892	Negative regulation of transcription, DNA-templated	14	ZNF282, ZHX2, TP63, ZHX3, CTCF, ZNF253, LGR4, SIRT2, GAS6, GZF1, PIAS4, IRF1, LIMD1, CCAR2	3.26644E-02
GO:0000978	RNA polymerase II core promoter proximal region sequence-specific DNA binding	11	JDP2, MAFB, GMEB1, IRF1, ZNF732, CTCF, NEUROG3, MAFA, ZNF253, GZF1, CRX	3.56281E-02
hsa04060	Cytokine-cytokine receptor interaction	8	IL18R1, TNFRSF1B, CCR6, IL2RA, FASLG, TNFRSF14, EDAR, CCL5	3.92933E-02
GO:0048388	Endosomal lumen acidification	2	CLCN3, FASLG	4.33113E-02
GO:0034112	Positive regulation of homotypic cell-cell adhesion	2	ANK3, CCL5	4.33113E-02
GO:0008283	Cell proliferation	11	TUSC2, ZMYND11, GLUL, SBDS, SLC29A2, IL2RA, GAB1, CDK11B, PIM2, RAPGEF3, GAS6	4.39472E-02
hsa05205	Proteoglycans in cancer	7	WNT2, CTTN, GRB2, ANK3, GAB1, HBEGF, FASLG	4.65064E-02

## Discussion

In this study, we revealed the tsRNAs transcriptional profiles in RHD with AF. We identified 77 markedly dysregulated tsRNAs (6 up-regulated and 71 down-regulated) in RHD with AF compared with RHD without AF. Bioinformatics analysis uncovered the altered biological functions, including regulation of transcription, DNA binding, intracellular, and cytokine-cytokine receptor interaction. These results aimed to explore the regulatory role of tsRNAs in RHD with AF, which drew more attention from other researchers toward conducting a further experiment on tsRNAs.

Identifying the non-coding RNAs changes profiles of serum (37), atrial appendages (38), atrium samples (39), and aortic valve (40) in RHD with AF has been well-documented in recent years. In this article, we chose the cardiac papillary muscle as the experimental sample. Because resection of this tissue can cause no damage to the surrounding structure during MVR surgery. Meanwhile, RHD can cause mitral pathologic change due to thickening of the papillary muscles (41, 42). Therefore, the altered non-coding RNAs profiles of papillary muscle might elucidate the pathological process of RHD.

Currently, high-throughput sequencing and bioinformatics analysis, which may put a deep insight into disease occurrence at the molecular level, are frequently used by scientists. Many studies have discovered that non-coding RNAs are dysregulated in RHD with AF (37–40, 43, 44). Previous research focused on the aberrant non-coding RNAs in disease due to their disease-specific expression profiles (38, 45). tsRNAs are abundant small non-coding RNA, constituting 4–10% of all cellular RNA (21). They are the fundamental components of the translation machinery. They deliver amino acids to the ribosome to translate the genetic information in an mRNA template into a corresponding polypeptide chain. Although the regulation of tsRNAs is similar to miRNAs regarding the related physiological and pathological processes, the higher stability and expression levels of tsRNAs place them as ideal biomarkers for diagnosing and prognosis in diseases (46). Recently, correlations between dysregulated tsRNAs expression and disease development have been reported (46). One of the principal drivers of the current tsRNA research is the discovery of abundant tsRNAs and tsRNA in mice and humans (47, 48). Additionally, their capabilities as a potential biomarker for disease diagnosis and prognosis have been revealed in clinical studies (46). Thus, it is worth identifying the dysregulation of tsRNAs in RHD with AF. In this study, we explored the tsRNAs expression profiles between RHD with AF and RHD without AF. The results indicated the length of tsRNAs was from 16 to 21 and 31 to 37 nucleotides (Figure 1C). The figures illustrated that the number, expression level, and type of tsRNAs have changed in the AF group (Figures 1, 2). All the results suggested the tsRNAs may be potential candidates for the pathophysiological process of AF.

Based on bioinformatics analysis, prior research demonstrated dilated cardiomyopathy, hypertrophic cardiomyopathy (38), and metabolic pathway (37) are the most important biological process of RHD with AF. In our research, one crucial pathway was enriched: cytokine-cytokine receptor interaction from KEGG pathway analysis. Cytokines and their receptor networks are an essential component of the body's signal transduction system (49). Cytokines can be widely involved in almost all physiological and pathological states of the body, affecting gene expression, cell membrane permeability, biological enzyme activity, and cytoskeletal protein function, leading to various physical effects on cells. In our study, eight target genes were enriched in cytokine-cytokine receptor interaction involving IL18R1, TNFRSF1B, CCR6, IL2RA, FASLG, TNFRSF14, EDAR, CCL5. CCL5 has been shown to orchestrate the recruitment to inflammatory sites of several inflammatory cell subsets, such as monocytes, neutrophils, dendritic cells, and lymphocytes through the binding to CCR1, CCR3, or CCR5 (50). CCL5 is increased in atherosclerosis (51), Myocardial infarction (50), and RHD (52). Treatment with anti-CCL5 mAb exerted cardioprotective effects (50). Nevertheless, no documents have addressed the relationship between CCL5 and AF. In the present research, CCL5 is a target gene of AS-tDR-001363, which is down-regulated in RHD with AF group. We overexpressed AS-tDR-001363 in AC16 cells to identify the corresponding alterations in tsRNA target genes. Although the tsRNAs mimics could not represent the actual tsRNAs, at present, mimics are usually used to explore the impact of tsRNAs on target genes (23, 27, 53). After transfection with AS-tDR-001363 mimics, the expression of CCL5 was predominantly downregulated (Figure 4B). Therefore, our study may provide a novel perspective to treat RHD with AF.

Overall, the research firstly shows the altered expression patterns of tsRNAs in RHD with AF. Given the pathogenesis and prognosis of diseases, we chose myocardial papilla to reveal more regulator function of tsRNAs in RHD with AF. What's more, the validation of tsRNAs function is still primarily needed by future researchers and the future studies with larger sample sizes will be needed to confirm our present results.

## Data Availability Statement

The raw data of tsRNAs-sequencing in this article will be acquired from GEO (https://www.ncbi.nlm.nih.gov/geo/query/acc.cgi?acc = GSE185581).

## Ethics Statement

The studies involving human participants were reviewed and approved by Ethic Committee of the Xiangya Hospital Central South University. The patients/participants provided their written informed consent to participate in this study.

## Author Contributions

YW and WL designed the experiments. Z-yY wrote the manuscript, analyzed the data, and visualized the figures. Z-qL and P-fL performed the experiments. TT revised the manuscript. All authors approved the submitted version.

## Funding

The Outstanding Youth Foundation of Hunan Provincial Natural Science Foundation of China (Grant No. 2019JJ30042), Innovation-Driven Project of Central South University (2020CX047), and the science and technology innovation Program of Hunan Province (2021RC3030) funded the project.

## Conflict of Interest

The authors declare that the research was conducted in the absence of any commercial or financial relationships that could be construed as a potential conflict of interest.

## Publisher's Note

All claims expressed in this article are solely those of the authors and do not necessarily represent those of their affiliated organizations, or those of the publisher, the editors and the reviewers. Any product that may be evaluated in this article, or claim that may be made by its manufacturer, is not guaranteed or endorsed by the publisher.
