# Supplementary figures and images for: Altered Expression of Transfer-RNA-Derived Small RNAs in Human With Rheumatic Heart Disease
Source: Front Cardiovasc Med. 2021 Dec 1;8:716716. doi: 10.3389/fcvm.2021.716716 (PMC8671610; doi:10.3389/fcvm.2021.716716)

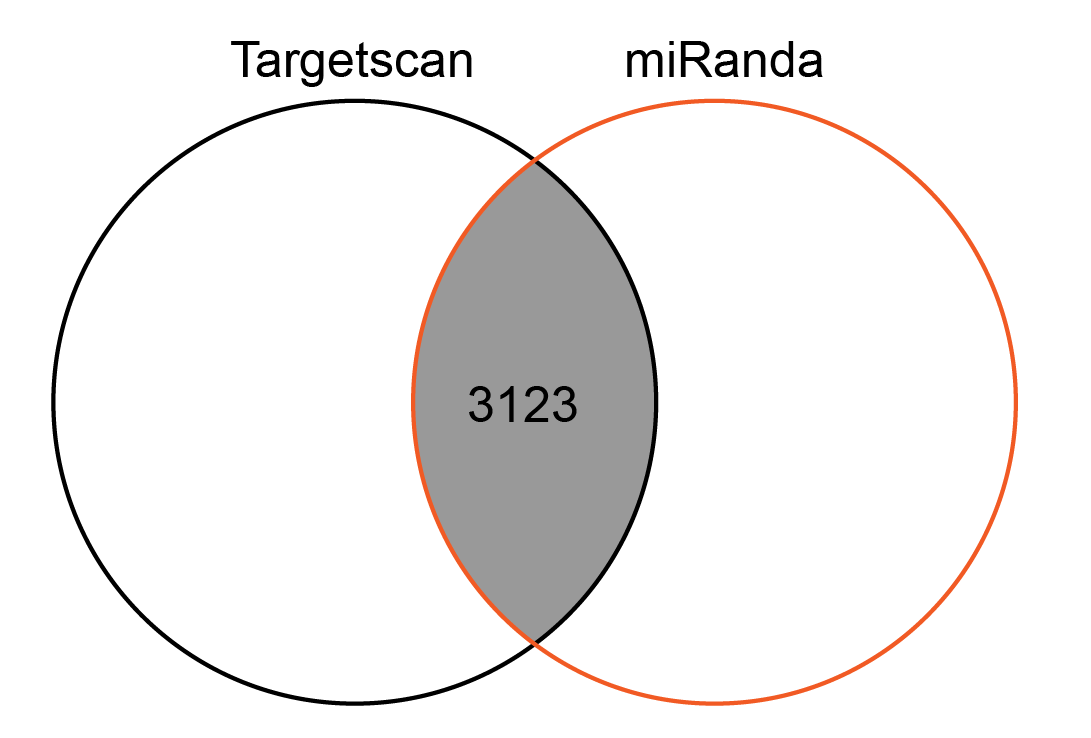

Supplement: Supplementary file 1 [file Image_1.tif]
